# Closing the value-based circle in shared decision-making: a digital framework for informing the shared decision-making process through patient reported outcome and experience measures

**DOI:** 10.3389/fpubh.2024.1452440

**Published:** 2024-08-29

**Authors:** Marta del Olmo Rodríguez, Rafael Martos Martinez, Adriana Pascual Martínez, Carolina Miranda Castillo, Jorge Short Apellaniz, Bernadette Pfang, Enrique Baca-García, Raúl Córdoba Mascuñano

**Affiliations:** ^1^Hospital Management, Quirónsalud 4H Network, Madrid, Spain; ^2^Clinical and Organizational Innovation Unit (UICO), Quirónsalud 4H Network, Madrid, Spain; ^3^Instituto de Investigación Sanitaria – Fundación Jiménez Diaz (IIS-FJD), Madrid, Spain; ^4^Department of Hematology, General Villalba University Hospital, Madrid, Spain; ^5^Department of Medicine, Universidad Europea de Madrid, Madrid, Spain; ^6^Infanta Elena University Hospital, Madrid, Spain; ^7^Department of Hematology, Infanta Elena University Hospital, Madrid, Spain; ^8^Department of Hematology, Rey Juan Carlos University Hospital, Madrid, Spain; ^9^Department of Psychiatry, Fundación Jiménez Díaz University Hospital, Madrid, Spain; ^10^Department of Medicine, Universidad Autónoma de Madrid, Madrid, Spain; ^11^Fundación Jiménez Díaz University Hospital, Madrid, Spain

**Keywords:** value-based healthcare, patient-reported outcomes, PROMS, digital patient decision aid, PREMs, shared decision-making

## Abstract

**Background:**

The spreading adoption of value-based models of healthcare delivery has incentivized the use of patient-reported outcomes and experience measures (PROMs and PREMs) in clinical practice, with the potential to enrich the decision-making process with patient-reported data.

**Methods:**

This perspective article explores PROs and the shared decision-making (SDM) process as components of value-based healthcare. We describe the potential of PROMs and PREMs within the decision-making process and present a digital framework for informing the shared decision-making process using aggregated data from a healthcare system PROMs and PREMs program, including early results from implementation in hospital network in Madrid, Spain.

**Results:**

The proposed digital framework incorporates aggregated data from a hospital network PROMs and PREMs program as part of a digital patient decision aid (PDA) for patients with lymphoma. After the first hematologist appointment, participating patients access the PDA to review relevant information about clinical and patient-reported outcomes for each of the possible options, assign a personal order of priority to different outcomes, and then select their preferred course of action. Patients’ answers are automatically uploaded to the EHR and discussed with hematologists at the next appointment. After beginning treatment, patients are invited to participate in the network PROMs program; participants’ PROMs data are fed back into the PDA, thus “closing the circle” between the decision-making process and patient-reported data collection.

During the first 14 months after launching the decision aid in October 2022, of 25 patients diagnosed with follicular lymphoma at the four participating hospitals, 13 patients decided to participate. No significant differences in age or sex were observed between groups. Average SDM Q-9 score for patients filling in the questionnaire (*n* = 6) was 36.15 of 45 points.

**Conclusion:**

Various obstacles toward widespread implementation of SDM exist such as time constraints, lack of motivation, and resistance to change. Support and active engagement from policy makers and healthcare managers is key to overcome hurdles for capturing patient-reported data and carrying out shared decision-making at healthcare system level. Early results of a digital framework for PRO-enriched SDM seem to be beneficial to the decision-making process.

## Introduction

The aim of value-based care is to deliver the highest value to patients at the lowest possible cost (1). In order to ensure value delivery, however, patient outcomes must be reported and measured consistently. Although important, traditional clinical outcomes (such as test results or survival) are not the only aspects of care in the value equation. In fact, there is often poor correlation between the information that patients report and the data which clinicians perceive as relevant and choose to record (2–4). To bridge this gap, patient-reported outcomes and experience measures (PROMs and PREMs) have been developed as standardized questionnaires which allow patients to report their experiences and outcomes during the care process in an objective, quantifiable way (5).

On the other hand, due to continuous advances in biomedical science, there are often several valid alternatives when faced with a diagnostic or therapeutic decision, ranging from aggressive and expensive therapies to “watchful waiting.” With the advent of patient autonomy, the paradigm of clinical decision making has shifted toward “shared decision-making” (6), in which both patients and healthcare professionals share the burden of making choices based on individual patients’ values and preferences as well as available scientific evidence.

The spreading adoption of value-based models of healthcare delivery has incentivized the use of PROMs and PREMs in clinical practice, with the potential to enrich the decision-making process with patient-reported data (7). However, various obstacles exist such as time constraints, lack of motivation, and resistance to change (8, 9). Support and active engagement from policy makers and healthcare managers is key to overcome hurdles for capturing patient-reported data and carrying out shared decision-making at healthcare system level (10). This perspective article explores patient-reported outcomes and the shared decision-making process as components of value-based healthcare, describes the potential of PROMs and PREMs within the decision-making process, and present a framework for informing the shared decision-making process using aggregated data from a healthcare system PROMs and PREMs program.

## What is value in healthcare, and why is measuring patient-reported outcomes important?

First described by Porter as a strategy to fix the current problems of healthcare systems worldwide, value-based healthcare is a model of care delivery that seeks to provide the highest value for patients while minimizing costs (1). Value is a multifaceted concept that entails providing meaningful outcomes. While some aspects of value, such as survival, are easily understood and measured, other outcomes – such as quality of life – are more elusive. Although these components of value are harder to define and quantify, they are of vital importance to patients. However, comprehensive information about multilevel consequences of available treatment options is seldom presented to patients during the decision-making process, mostly due to lack of scientific evidence, as patient-reported outcomes have often been ignored when designing protocols for clinical trials (11).

To bridge the existing knowledge gap regarding relevant healthcare outcomes, over the last decade two decades patient reported outcomes and experience measures (PROMs and PREMs) have been developed and validated as psychometrically robust tools to collect information on patients’ assessment of clinical and non-clinical aspects of care (5). PROMs and PREMs can be either general or condition-specific, such as the PREM-ECM questionnaire for patient experience with experimental treatments in cancer (12), or the Minnesota Living with Heart Failure Questionnaire which measures physical, socioeconomic, and emotional aspects of quality of life for patients with heart failure (13). Capturing PROMs and PREMs has demonstrated multiple benefits, with higher patient satisfaction, better communication between patients and clinicians, and improved symptom control observed in patients participating in patient-reported outcome programs (14–16). However, despite the development of different strategies to implement PROMS and PREMs in clinical practice, and the increasing use of patient-reported outcomes as endpoints for clinical trials, widespread implementation remains a challenge due to various factors (17). Clinicians may not understand the importance of patient-reported data for delivering quality care, while patients may lack time and motivation to complete questionnaires. Furthermore, many healthcare systems lack sufficient technological infrastructure to analyze aggregated patient-reported data. Some reports of digital programs for collecting patient-reported data over a web application have demonstrated high response rates (18), and strategies aimed at patient and healthcare professional education about the importance of PROMs and PREMs for improving health results have shown to improve participation (19). The role of policy makers and health care managers is crucial to design, implement and monitor PROMs and PREMs initiatives in order to achieve the goal of measuring outcomes for every patient and delivering true value in healthcare.

## Practical applications of PROMs and PREMs in clinical practice: shared decision-making

While the importance of collecting patient-reported data has been the topic of much research, in the current era of big data a question which policymakers and healthcare managers should ask themselves is how to use aggregated patient reported data to improve value delivery. Some systems have used results from PROMs and PREMs programs to inform value-based purchasing (20) or to modify the way in which care is organized (19). The clinical decision-making process is another area in which data could be harnessed in order to provide patients with accurate information, not only about traditional results of treatment such as survival (which is often the only information available), but also about other consequences of care which may be of equal or even more importance from the patient’s point of view (10, 21).

Shared decision-making is the process in which both patients and healthcare professionals participate in choosing a clinical course of action which takes into account both available scientific evidence and patients’ preferences and values (22, 23). Shared decision-making is a natural consequence of the recognition of patient autonomy, shifting away from the authoritarian model of doctor-patient relationship. The steps in the shared decision-making process include recognizing that a decision is required, presenting and understanding the best available evidence, and incorporating patients’ values and preferences into the decision (24). Although shared decision-making is at the heart of value-based care – providing value for patients entails considering patients’ individual values and preferences – in practice, implementing shared decision-making is difficult. Healthcare professionals find that lack of time is the main obstacle toward shared decision-making (9). Patient decision aids can help to facilitate the shared decision-making process by providing a starting point for discussion about available options and consequences and have shown to reduce decisional conflict and improve patient participation in making clinical choices (25–28). However, they are not a standalone solution for obstacles of shared decision-making, as they cannot replace the dialog between patients and clinicians. At the same time, patient decision aids could be a unique opportunity to present data from PROMs and PREMs programs and incorporate patient reported data into clinical decisions.

Policy makers and healthcare managers have an important role to play in facilitating shared decision-making by stressing the importance of patient participation in clinical decisions, highlighting the aim of healthcare delivery as providing value for patients instead of delivering lower-value care to a higher volume of patients. As lack of time is the number one obstacle to shared decision-making for healthcare professionals, managers should take an active role in transforming clinical processes to “make room” for decision-making in which unhurried dialog between patients and their doctors is a fundamental component. At the same time, support from healthcare managers is crucial if systems are to leverage aggregate data from PROMs and PREMs programs in order to inform the decision-making process. In the next section, we present a digital framework for shared decision-making using PROMs data to improve decisional quality by informing patient choices, while providing support to patients and clinicians by creating a unique “space” for decision-enabling dialog to take place.

## A digital framework to inform the shared decision-making process through aggregated PROMs and PREMs

### Setting and participants

The digital framework for shared decision-making was designed and implemented at four public hospitals (Fundación Jiménez Díaz University Hospital, Infanta Elena University Hospital, Rey Juan Carlos University Hospital, and General Villalba University Hospital) outsourced to a value-based healthcare network (Quirónsalud) in Madrid, Spain, and covering a catchment area of more than one million inhabitants from the Madrid Health Service.

To design the framework, we decided to focus on the shared decision-making process in patients with a new diagnosis of follicular lymphoma, a high-impact clinical condition with multiple valid treatment options. Follicular lymphoma is the second most common subtype of non-Hodgkin lymphoma (29), with an estimated age-standardized incidence of 2–4 new diagnoses per 100,000 person-years (30–32). Due to its typically indolent nature, the optimal management of follicular lymphoma is unclear, with options ranging from different schemes of chemotherapy (rituximab, bendamustine plus rituximab, or R-CHOP) to watchful waiting (33). At the four participating centers, a total of 20–30 new cases of follicular lymphoma are diagnosed each year.

A multidisciplinary team, including hematologists, oncologists, hospital managers, patient experience staff, and members of the information systems department, was formed to design and develop the framework. Over a series of meetings from January 2022 to June 2022, an initial framework was designed and validated by members of the hematology departments from the four participating centers. The project was implemented and launched in October 2022.

### Framework design and development

#### System prerequisites

To ensure success, several prerequisites exist for a digital framework aiming to enrich the shared decision-making process with aggregated PROMs data, including the existence of a system-wide electronic health record and a fully implemented data collection program for patient-reported outcomes. The four participating centers have used the Casiopea® (Inetum, Saint-Ouen, France) EHR since 2015. Patients can access their individual EHR through a smartphone via a web application, the Patient Portal, which also acts as an interface for other functions such as communicating with the care team, learning about different clinical conditions through interactive educational programs, and answering health questionnaires (34).

The network’s digital PROMs and PREMs program, E-Res Salud (19), covers more than 15 clinical conditions from different medical and surgical specialties including hematological malignancies. For patients with lymphoma choosing to participate, PROMs and PREMs questionnaires are automatically sent out over the Patient Portal at predefined timestamps (before treatment (baseline); and at 3, 6, 9 and 12 months after starting treatment). These questionnaires include the EuroQol-5D, HM-PROM, and PRO-CTCAE instruments. Results from the questionnaires are automatically saved to each individual patient’s EHR as well as to the E-Res Salud database. Clinicians and healthcare managers can view, export, and analyze aggregated data from the E-Res Salud database using Microsoft PowerBI® (Microsoft Corporation, Redmond, Washington) dashboards.

#### Integrating digital and face-to-face care in shared decision-making

The shared decision-making process is, as its name implies, a process that involves multiple interactions between patients and clinicians. While a digital infrastructure can improve aspects such as access to information, efficiency, and traceability, the importance of face-to-face communication is crucial. To empower the decision-making process with a digital framework, we decided to designate two specific in-person checkpoints for clinicians and patients: realizing that a decision must be made (and that several alternatives exist), and making the decision itself. These checkpoints take place at two different appointments at the hematology outpatient clinic. At the first appointment, which coincides with a new diagnosis of follicular lymphoma, the treating clinician explains that a decision must be made regarding treatment options, presents the different options for treatment, and offers patients the possibility of participating in the shared decision-making program. If the patient agrees, the clinician activates access to a digital patient decision aid (Decide Salud™), as well as ordering standard tests to complete lymphoma staging as part of usual care. At the second appointment, the patient and clinician review the patient’s answers, along with any doubts or concerns, and a choice of treatment is made. It is important to note that starting treatment is not delayed because of choosing or declining to participate in the program.

**Figure 1:**
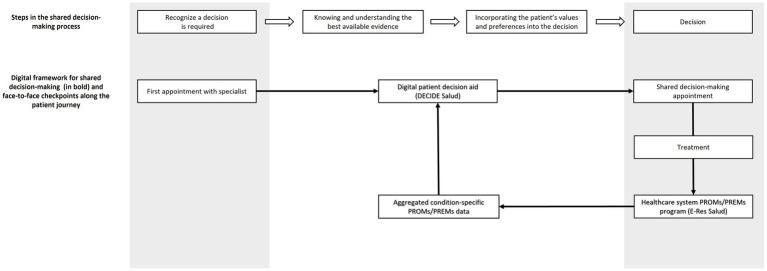
Elements of a digital framework (in bold) for shared decision-making incorporating aggregated PROMs and PREMs from a network database, and integrated with face-to-face checkpoints along the patient journey. After beginning treatment, patients are invited to participate in the E-Res Salud program; participants’ PROMs and PREMs data is fed back into the PDA, thus “closing the circle” between the decision-making process and PROMs and PREMs data collection.

The digital framework incorporates PROMs data from the E-Res Salud database taken from patients diagnosed with lymphoma between 2019 and 2022, integrating these data as part of a digital patient decision aid (PDA) to help patients understand the different available treatment choices and align the choice of treatment with their personal hierarchy of values and preferences. After the first appointment, participating patients access the PDA over the Patient Portal web application to review relevant information about clinical and patient-reported outcomes for each of the possible options (including watchful waiting), assign a personal order of priority to different outcomes, and then select their preferred course of action. Patients’ answers are automatically uploaded to the EHR, and treating clinicians also receive an automated report with their patients’ answers to review before the second checkpoint appointment. After beginning treatment, patients are invited to participate in the E-Res Salud program; participants’ PROMs and PREMs data is fed back into the PDA, thus “closing the circle” between the decision-making process and PROMs and PREMs data collection (Figure 1).

#### Developing the Decide Salud^TM^ PDA

To develop the Decide Salud™ PDA, we extracted aggregated PROMs data from the E-Res Salud database for all lymphoma patients diagnosed at the four participating hospitals between 2019 and 2022 (*n* = 173). We organized the PROMs data for each treatment option based on the five essential dimensions of quality of life and symptoms defined by the European Hematology Association, defined by a multidisciplinary group including patients and patient advocacies. These dimensions include emotional status, physical activity, eating habits, social life, and long-term perception of symptoms and/or toxicity. Data were expressed as the percentage of patients reporting high or very high impact for a specific dimension of quality of life and symptoms, following International Patient Decision Aid Standards (IPDAS) quality criteria (35). New PROMs data from the E-Res Salud database were incorporated on a bi-annual basis.

The PDA also incorporated information on clinical outcomes for each treatment option. Information on clinical outcomes was structured in two categories: treatment effectiveness and survival, and treatment characteristics. Data for treatment effectiveness and survival for each alternative were based on available evidence from five-year follow-up results of a randomized clinical trial comparing different treatment options (33).

**Figure 2:**
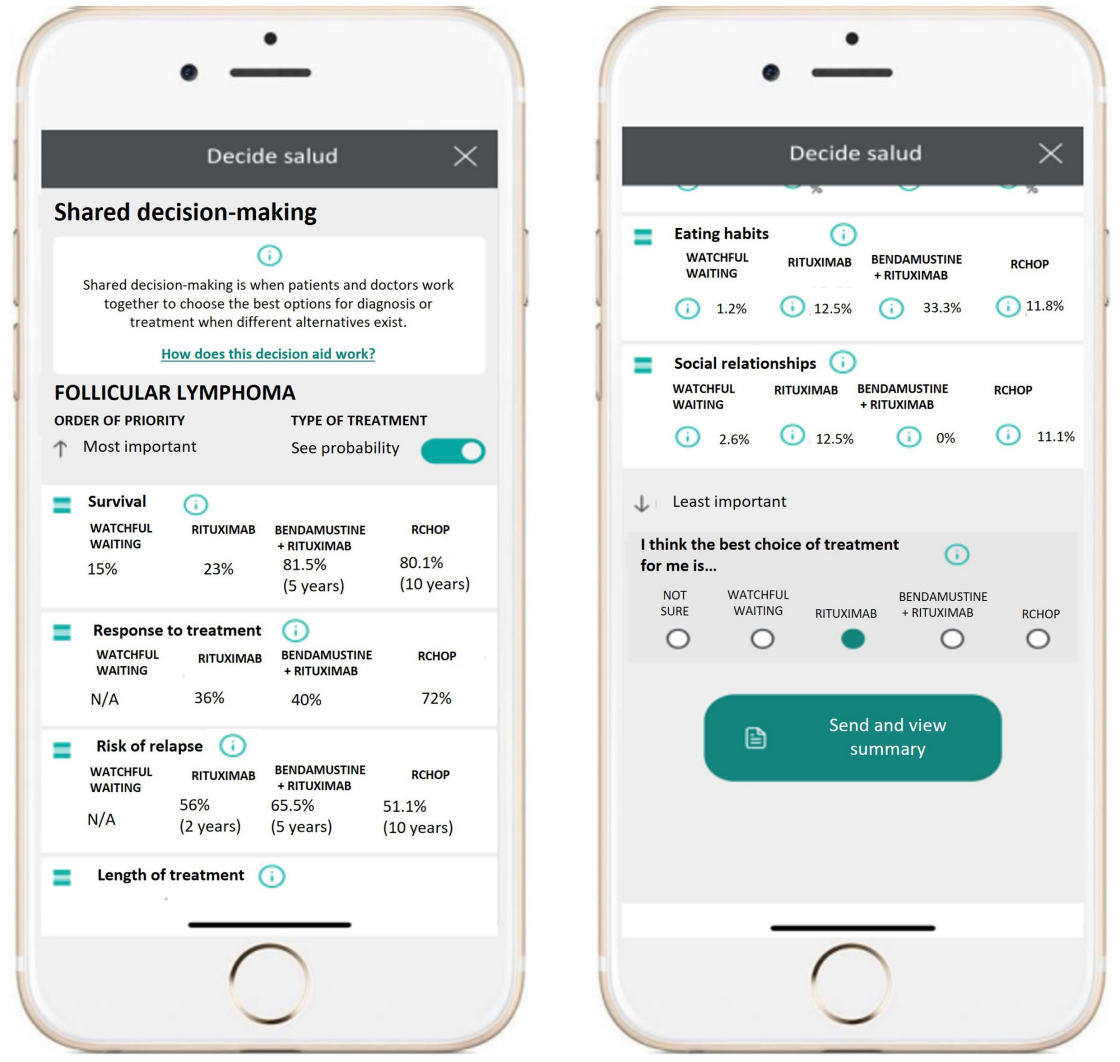
Patient interface for the DECIDE Salud digital patient decision aid.

With the aid of members of the information systems department, the PDA was integrated with the network’s electronic health record (Casiopea®, Inetum) and an easy-to-operate web interface was designed for patient access through the electronic patient portal (Figure 2). A drag-and-drop design permitted patients to list clinical and patient-reported outcomes in order of relative importance. Hovering icons were designed to provide full information on different clinical and patient-reported outcomes for each treatment option while avoiding visual fatigue. Patients choosing to participate were asked to review a list of relevant clinical outcomes and patient-reported outcomes, such as survival, amount of time spent at the hospital, physical activity, and emotional status and sort them by order of relative importance using a “drag and drop” function. Patients were then presented with the available treatment options (including abstaining from treatment) and user-friendly information about the impact of each option on the different outcome and experience variables, with frequency expressed as a range of percentages. Participants were encouraged to review their preferences as many times as necessary to feel ready to select the treatment option that best fitted their values and priorities. Before selecting a treatment option, patients were reminded that their selection was not binding in any way, and that they would have the opportunity to go over any questions or doubts with their physician at the next appointment. Once a treatment preference was selected, the DECIDE Salud decision aid generated a digital report summarizing the patient’s choice and scale of priorities. The report was automatically uploaded to the patients’ electronic health record for review by the hematology consultant prior to the decision-making appointment.

During follow-up appointment, patients were encouraged to discuss their preferred treatment option, and to voice any concerns or questions about aspects of therapy mentioned in the decision aid, or any other doubts they might have. Finally, the clinical course of action was decided upon, and patients were included in the appropriate clinical pathway to receive the chosen treatment. Patients were also asked to complete the patient version of the SDM-9-Q questionnaire (36) to assess the quality of shared decision-making. This questionnaire is a validated instrument featuring 9 items measuring the quality of the decisional process (Supplementary Table S1).

### Initial experience

We decided to consolidate our SDM framework as a 16-month pilot project (from October 2022 to February 2024), developing a specific clinical pathway for selected patients and deploying the digital patient decision aid, DECIDE Salud, to ensure that patients faced with the decision-making process benefit from available patient reported outcomes evidence from the network PROMs program, combined with relevant information from scientific literature, transmitted via a user-friendly interface. Patients were selected for the SDM pilot project based on a recent diagnosis of follicular lymphoma, full cognitive capacity, and willingness to participate. On their first specialist appointment with a hematology consultant, patients agreeing to participate were presented with the different options for treatment and registered for the DECIDE Salud decision aid by the hematologist. After registration, access to the decision aid was activated on the patient’s EHR-linked webapp. A follow-up appointment was made to review the decision-making process, to go over any questions, concerns, or doubts that the patient might have regarding treatment options, and to choose the most appropriate course of action.

During the first 16 months after launching the decision aid, of 25 patients diagnosed with follicular lymphoma at the four participating hospitals, 13 patients decided to participate in the DECIDE Salud initiative. Seven participants in both groups were women. In the study group, patients presented a median age of 55 years (range 35–79, IQR 23), while median age for the control group was 64 years (range 30–78, IQR 14), with no statistically significant differences observed between groups using the Mann–Whitney U test. Average SDM Q-9 score for patients filling in the questionnaire (*n* = 6) was 36.15 of 45 points. Individual scores for each of the 9 items of the survey are presented in Table 1. Clinicians choosing to participate in the initiative reported high satisfaction with the decision-making process.

**Table 1:** Individual answers for the nine items of the SDM-Q-9 questionnaire from patient participating in the DECIDE Salud program, along with their demographic and clinical characteristics.

Patient	Demographic and clinical characteristics				SDM-Q-9 Items									
	Sex	Age	Presenting complaint	Course of action	My doctor made clear that a decision needs to be made.	My doctor wanted to know exactly how I want to be involved in making the decision.	My doctor told me that there are different options for treating my medical condition.	My doctor precisely explained the advantages and disadvantages of the treatment options.	My doctor helped me understand all the information.	My doctor asked me which treatment option I prefer.	My doctor and I thoroughly weighed the different treatment options.	My doctor and I selected a treatment option together.	My doctor and I reached an agreement on how to proceed.	Total SDM-Q-9 score
1	Female	45	Adenopathy	Start a new treatment	Strongly agree	Strongly agree	Strongly agree	Strongly agree	Strongly agree	Somewhat agree	Somewhat agree	Strongly agree	Strongly agree	34
2	Male	35	Fever	Start a new treatment	Completely agree	Completely agree	Completely agree	Completely agree	Completely agree	Completely agree	Completely agree	Completely agree	Completely agree	45
3	Male	80	Submandibular mass	Start a new treatment	Completely agree	Completely agree	Completely agree	Completely agree	Completely agree	Completely agree	Completely agree	Completely agree	Completely agree	45
4	Female	69	Accidental finding	Watchful waiting	Completely agree	Completely agree	Strongly agree	Strongly agree	Strongly agree	Strongly agree	Strongly agree	Strongly agree	Strongly agree	38
5	Female	56	Ocular mass	Continue a previous treatment (relapse)	Strongly agree	Completely agree	Completely agree	Completely agree	Completely agree	Completely agree	Completely agree	Completely agree	Completely agree	44
6	Female	56	Adenopathy	Watchful waiting	Somewhat disagree	Completely agree	Completely agree	Completely agree	Completely agree	Somewhat disagree	Completely agree	Completely agree	Completely agree	39

## Discussion

Several studies acknowledge the potential of PROMs and PREMs data as a way of improving the decision-making process by providing information, not only about clinical outcomes such as survival, but also about outcomes that have historically been underrepresented in the scientific literature, such as the impact of different treatment options on social life, eating habits, and emotional status (13, 21, 37–39). However, obstacles such as lack of time, lower levels of technological maturity, and resistance to change often make implementation difficult (40). Policy makers and healthcare managers should provide active support by allocating resources for patient and clinician education, as well as helping to develop tools and workflows geared toward facilitating the shared decision-making process. This article explores the concepts of patient-reported data and shared decision-making in the context of value-based healthcare and describes a digital framework for informing clinical decision-making using PROMs and PREMs. In this context, healthcare managers from a hospital network in Madrid, Spain, collaborated with clinicians and members of the information technology department to design a digital patient decision aid which incorporates aggregated PROMs data from the network PROMs and PREMs program, thus providing patients with information from other patients in a similar clinical context. The patient decision aid was incorporated as part of the patient care workflow and integrated with the EHR to enable oncologists to review patients’ input before coming to a final, shared clinical decision.

To the best of our knowledge, this is the first report of a digital patient decision aid for patients with follicular lymphoma. The innovative design of the DECIDE Salud decision aid fills a gap in the existing literature, as it demonstrates the successful use of aggregated PROMs to inform the decision-making process in patients with follicular lymphoma. Half of eligible patients chose to participate, and no significant differences in age or sex were observed between patients choosing to participate and non-participants, providing initial evidence that the tool is accessible across age groups. This evidence contrasts with findings from a recent study which demonstrates that older adults are at risk for lower access to health technology (41). A recent systematic review of decision aids in hematologic malignancies concluded that research on PDAs for this group of patients is limited (28). Although existing evidence points to an association between PDAs and increased patient knowledge (42–44), DECIDE Salud is the first program for patients with lymphoma to assess the quality of the shared decision-making process using validated tools such as the SDM-Q-9 or the OPTIONS scale.

Our study has several limitations, including the small number of patients participating in the initial implementation of DECIDE Salud. The lack of direct patient involvement in the design of the decision aid is a limitation of the framework’s design. However, when designing the content of the patient decision aid, we based the structure of the information provided on European Hematology Association criteria, which draw on the inputs of patients and patient advocacies. Also, we did not include specific feedback from participating hematologists using the SDM-Q-Doc, the clinician version of the SDM-Q-9. Further research is needed to confirm the generalizability of our findings as well as to explore the impact of the DECIDE Salud program on clinical and patient-reported outcomes.

## Conclusion

Collecting patient-reported data through PROMs and PREMs (patient reported outcome and experience measures) to measure outcomes which truly matter to patients is at the heart of value-based healthcare reform. Shared decision-making should - in theory - be informed by patient-reported data. However, in practice, the integration of PROMs and PREMs in the shared decision-making process remains elusive, due in part to health systems failing to leverage digital transformation in this area. Policy makers and healthcare managers play a critical role in encouraging the use of PROMs and PREMs and promoting shared decision-making. The digital framework we propose integrates PROMs and PREMs into the decision-making process to ensure that clinical decisions integrate not only traditional clinical outcomes but also data on outcomes which truly reflect patients’ values and preferences.

## Data Availability

The raw data supporting the conclusions of this article will be made available by the authors, without undue reservation.
