# Supplementary material for: Closing the value-based circle in shared decision-making: a digital framework for informing the shared decision-making process through patient reported outcome and experience measures
Source: Front Public Health. 2024 Aug 29;12:1452440. doi: 10.3389/fpubh.2024.1452440 (PMC11390557; doi:10.3389/fpubh.2024.1452440)
Supplement: Supplementary file 1 [file Table_1.DOCX]

**SDM-Q-9 Items**

**Patients are instructed to select one of the following options: completely disagree (0), strongly disagree (1), somewhat disagree (2), somewhat agree (3), strongly agree (4), or completely disagree (5).**

1. My doctor made clear that a decision needs to be made.
2. My doctor wanted to know exactly how I want to be involved in making the decision.
3. My doctor told me that there are different options for treating my medical condition.
4. My doctor precisely explained the advantages and disadvantages of the treatment options.
5. My doctor helped me understand all the information.
6. My doctor asked me which treatment option I prefer.
7. My doctor and I thoroughly weighed the different treatment options.
8. My doctor and I selected a treatment option together.
9. My doctor and I reached an agreement on how to proceed.
